# Ectopic Lingual Thyroid

**DOI:** 10.1155/2015/252357

**Published:** 2015-03-29

**Authors:** Khaled Khamassi, Habib Jaafoura, Fahmi Masmoudi, Rim Lahiani, Lobna Bougacha, Mamia Ben Salah

**Affiliations:** Department of Otorhinolaryngology-Head and Neck Surgery, Charles Nicolle Hospital, Boulevard 9 Avril, 1006 Tunis, Tunisia

## Abstract

Ectopy of the thyroid gland is an abnormal embryological development. Its occurrence in children is rare. In this study, we report the case of a 12-year-old girl that presented with dysphagia and nocturnal dyspnea. Magnetic resonance imaging confirmed the presence of a lingual thyroid. Thyroid scintigraphy showed intense and elective uptake of radiotracer at the base of the tongue. Hormonal tests revealed hypothyroidism. Treatment consisted of opotherapy based on levothyroxine. Evolution has been favourable and the patient showed significant improvement with reduction of the dyspnea and the dysphagia and normalization of thyroid hormone tests.

## 1. Introduction

Ectopy of the thyroid gland is an abnormal embryological development, defined by an aberrant localization of thyroid tissue outside the thyroid compartment. It is a very rare entity. Its frequency is estimated at 1/4000 to 1/8000 among patients with hypothyroidism and 0.3% of all diseases of the thyroid gland. It particularly affects young women. Its occurrence in children is rare. Diagnosis is mainly based on clinical examination and imaging. Treatment is mainly medical and must take into account the physiological requirements for thyroid hormones.

In this study, we report a case of lingual thyroid and we review the literature on this topic.

## 2. Case Report

A 12-year-old girl without past medical history consulted for high dysphagia evolving for three years. Dysphagia had worsened over the past two months and was accompanied by increasing in nocturnal dyspnea and recent onset of sleep apnea. There were neither signs of thyroid dysfunction nor alteration of general condition.

Nasofibroscopy showed a reddish oval formation, with a diameter of 2 cm, located behind the lingual V and attached to the base of the tongue. At intraoral palpation, the mass was firm, smooth, uniform, and painless, with no bleeding. Examination of the neck revealed no palpable thyroid gland in the normal pretracheal position and no cervical lymphadenopathies.

**Figure 1:**
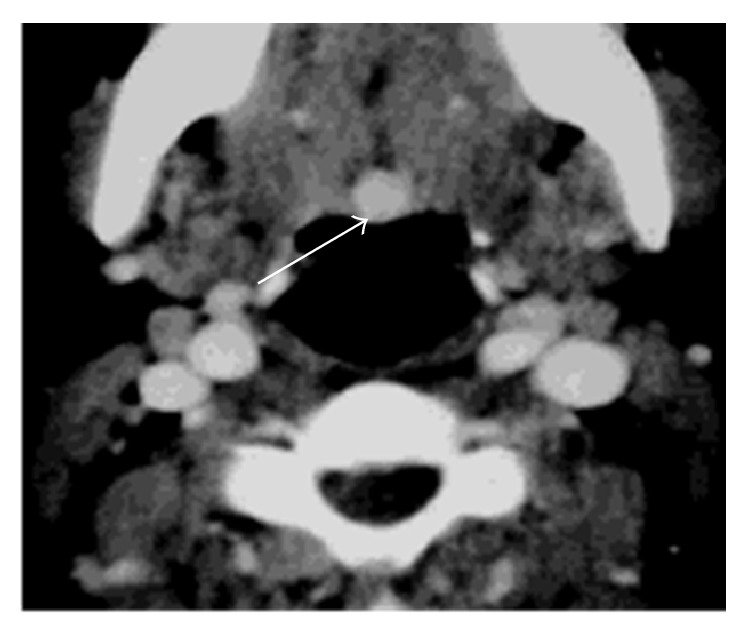
Axial CT: rounded lesion at the base of the tongue.

**Figure 2:**
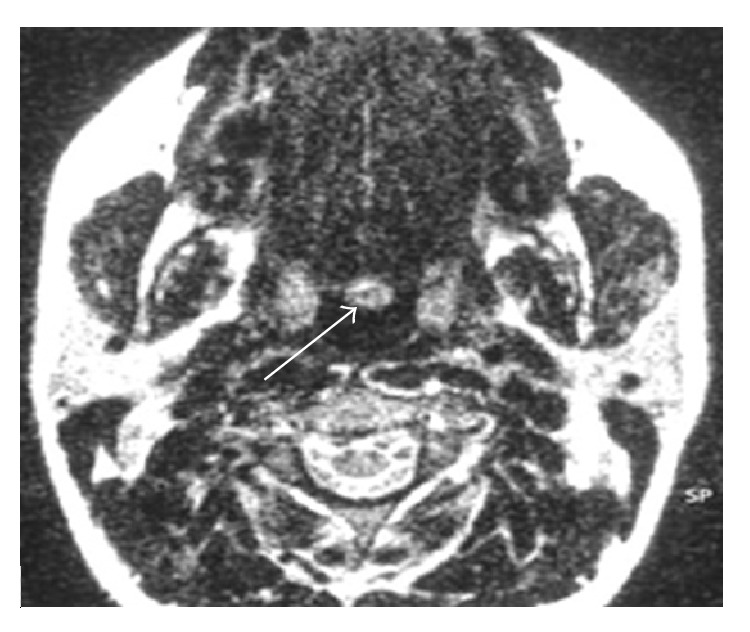
Axial MRI: lingual thyroid.

**Figure 3:**
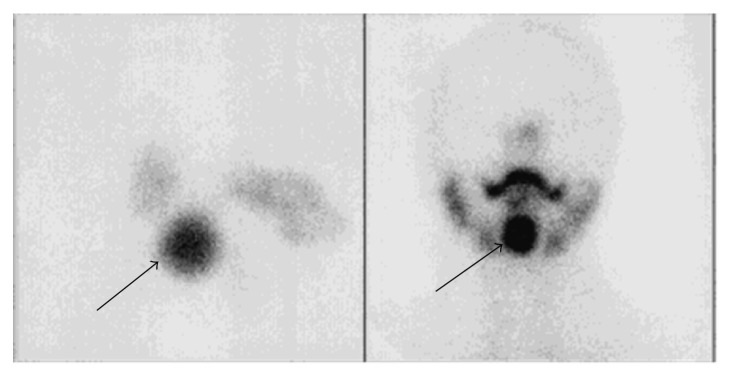
Thyroid scintigraphy: intense and elective uptake at the base of the tongue.

Cervical CT (Figure 1) scan showed a rounded lesion located at the base of the tongue, with heterogeneous enhancing after injection of iodine contrast medium. Thyroid compartment was empty. Magnetic resonance imaging confirmed the presence of a basilingual thyroid (Figure 2). Thyroid scintigraphy with technetium (Tc99m) showed intense and elective uptake of radiotracer at the area of the base of the tongue and no uptake in the normal thyroid location (Figure 3).

Hormonal tests showed subclinical hypothyroidism with a normal dosage of FT_4_ (15.4 pmol/L) and a slight increase of TSH (5.2 IU/mL).

**Figure 4:**
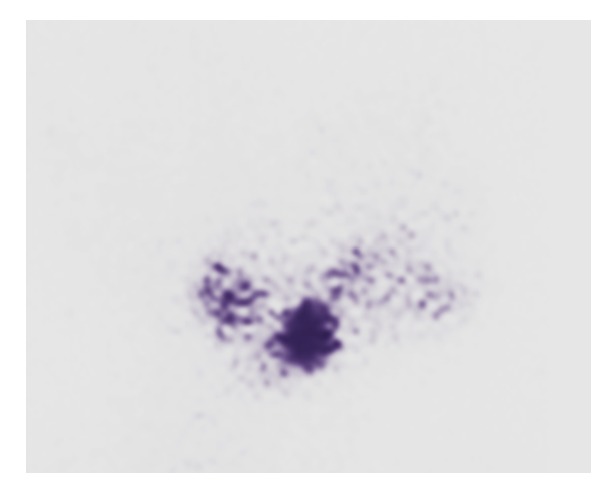
Posttherapeutic scintigraphy.

Treatment consisted of an opotherapy based on levothyroxine at the dose of 75 micrograms per day. Evolution has been favourable and the patient showed significant improvement in symptoms with reduction of the dyspnea and the dysphagia and normalization of thyroid hormone tests. Posttherapeutic thyroid scintigraphy showed a less intensive fixation at the base of the tongue compared to the initial scintigraphy (Figure 4).

## 3. Discussion

The thyroid tissue reaches the normal location in the pretracheal region by migrating caudally from the foramen cecum in the tongue base at the seventh week of fetal life. Ectopic lingual thyroid is caused by noncompletion of this migration [1, 2]. The normal thyroid gland can be seen together with ectopic thyroid tissues. A normally located thyroid is not seen in 70% of patients with lingual thyroid, as it was for our patient [3].

Lingual thyroid tissue is the most frequent ectopic location of the thyroid gland, although its clinical incidence is low with 1 in 100 000 cases occurring [4]. There are four groups of lingual thyroid: lingual, sublingual, thyroglossal, and intralaryngeal [5].

Lingual thyroid does not usually lead to any symptoms unless an increase in gland size occurs. In symptomatic cases, patients present with complaints of dysphagia, dysphonia, foreign body sensation in the throat, cough, pain, bleeding, and dyspnea [2, 5, 6]. Rarely, the lingual thyroid may cause hyperthyroidism [7] or be the site of thyroid cancer [8]. Endocrine changes such as puberty, pregnancy, and menstruation can lead to an increase in gland size and symptoms. This explains why lingual thyroid is 7-fold higher among women [3].

Biopsy is not recommended because of the risks of bleeding and infection [1, 9, 10]. On the ultrasonography, lingual thyroid is homogenous with regular contours and more echogenicity compared to tongue muscles [3]. Thyroid scintigraphy is indicated for the differential diagnosis of tongue base masses detected on physical examination or when thyroid tissue is not detected in the normal location on ultrasonography [11]. In our case, while the thyroid activity was not detected in the normal location, it was detected at the tongue base.

MRI provides valuable information about the exact size and location of the ectopic tissue and the presence of accompanying thyroglossal duct in patients for whom an operation is planned [3]. It is also helpful for determination of the posterior pharyngeal opening and the degree of narrowness in the cases with obstructive sleep apnoea syndrome (OSAS) [3, 11]. On MRI, lingual thyroid tissue is observed as either iso- or hypointense to the tongue muscles in T1-weighted sequences and more hyperintense than the tongue muscles in T2.

There are a few reports in the literature about benign oropharyngeal masses resulting in dysphagia and OSAS [12, 13]. Lipomas constitute the majority of these masses. Other masses include haemangioma, neurofibromas, and retention cysts. The gold standard test in the diagnosis of OSAS is polysomnography, with measure of the Apnoea-Hypopnoea Index [3, 12, 13].

The fact that the ectopic thyroid tissue can be the only functional thyroid tissue must be kept in mind when determining the therapeutic approach. Asymptomatic cases can be monitored with suppressive hormonal therapy aiming for reduction of ectopic tissue volume. Decrease in symptoms can occur with suppressive treatment in some cases [3]. Conservative treatment had proved its efficiency in many studies [3, 5, 9, 13, 14]. Indeed, administration of a suppressive dose of thyroid hormones aims to decrease the TSH level; therefore it can reduce the ectopic glandular volume and consequently reduce all the compressive symptoms.

Effective treatment for lingual thyroid is surgical excision, but no surgical treatment should be attempted until radioactive isotope scan has determined that there is an adequate thyroid tissue in the neck [14]. Surgical indications are important dyspnea or dysphagia, suspicion of malignancy, uncontrolled hyperthyroidism, and repetitive or severe bleeding. Transient tracheostomy may be required when surgery is indicated. Surgical excision can be made either transorally or externally with pharyngotomy through a transhyoidal approach [3, 14–16]. The surgeon has to perform meticulous homeostasis with bipolar electrocautery during tumor dissection to prevent postoperative haemorrhagic complications. Another method is transoral laser excision [17, 18]. Transoral radiofrequency ablation may also reduce the tissue volume [19]. In patients lacking thyroid tissue in the neck, lingual thyroid can be excised and autotransplanted to the muscles of neck [14, 20] or they may be put under lifelong postoperative hormone replacement therapy.

An alternative treatment for patients not accepting surgical treatment or for those not appropriate for general anaesthesia is radioactive iodine therapy [1, 3, 12]. This one is not recommended in cases where there is another functional thyroid tissue.

Surgical treatment, radioactive iodine ablation, CPAP, and intraoral devices used during sleep are the treatment options for cases with severe dyspnoea or OSAS [15]. CPAP usually provides a temporary improvement of the symptoms and is not well tolerated by patients in the long term [3].

## 4. Conclusion

When a mass lesion is observed in the tongue base, ectopic lingual thyroid must be taken into consideration in the differential diagnosis, and the diagnosis must be verified using ultrasonography, scintigraphy, CT scan, and MRI. Therapeutic approach should be considered according to symptomatology. The risks and benefits of each treatment modality should always be discussed with the patient.

## References

[1] Toso A., Colombani F., Averono G., Aluffi P., Pia F. (2009). Lingual thyroid causing dysphagia and dyspnoea. Case reports and review of the literature. *Acta Otorhinolaryngologica Italica*.

[2] Thomas G., Hoilat R., Daniels J. S., Kalagie W. (2003). Ectopic lingual thyroid: a case report. *International Journal of Oral and Maxillofacial Surgery*.

[3] Babademez M. A., Günbey E., Acar B., Günbey H. P. (2012). A rare cause of obstructive sleep apnea syndrome: lingual thyroid. *Sleep and Breathing*.

[4] Rabiei S., Rahimi M., Ebrahimi A. (2010). Coblation assisted excision of lingual thyroid. *Indian Journal of Otolaryngology and Head and Neck Surgery*.

[5] Chiu T.-T., Su C.-Y., Hwang C.-F., Chien C.-Y., Eng H.-L. (2002). Massive bleeding from an ectopic lingual thyroid follicular adenoma during pregnancy. *American Journal of Otolaryngology: Head and Neck Medicine and Surgery*.

[6] Gonciulea A., Cooper D. S., Salvatori R. (2014). Lingual thyroid. *Endocrine*.

[7] Abdallah-Matta M. P., Dubarry P. H., Pessey J. J., Caron P. (2002). Lingual thyroid and hyperthyroidism: a new case and review of the literature. *Journal of Endocrinological Investigation*.

[8] Massine R. E., Durning S. J., Koroscil T. M. (2001). Lingual thyroid carcinoma: a case report and review of the literature. *Thyroid*.

[9] Huang T. S., Chen H. Y. (2007). Dual thyroid ectopia with a normally located pretracheal thyroid gland: case report and literature review. *Head and Neck*.

[10] Hazarika P., Siddiqui S. A., Pujary K., Shah P., Nayak D. R., Balakrishnan R. (1998). Dual ectopic thyroid: a report of two cases. *Journal of Laryngology and Otology*.

[11] Giovagnorio F., Cordier A., Romeo R. (1996). Lingual thyroid: value of integrated imaging. *European Radiology*.

[12] Barnes T. W., Olsen K. D., Morgenthaler T. I. (2004). Obstructive lingual thyroid causing sleep apnea: a case report and review of the literature. *Sleep Medicine*.

[13] Taibah K., Ahmed M., Baessa E., Saleem M., Rifai A., Al-Arifi A. (1998). An unusual cause of obstructive sleep apnoea presenting during pregnancy. *The Journal of Laryngology & Otology*.

[14] Kumar S. S., Muthiah Selva Kumar D., Thirunavukuarasu R. (2013). Lingual thyroid-conservative management or surgery? a case report. *Indian Journal of Surgery*.

[15] Peters P., Stark P., Essig G. (2010). Lingual thyroid: an unusual and surgically curable cause of sleep apnoea in a male. *Sleep and Breathing*.

[16] Amr B., Monib S. (2011). Lingual thyroid: a case report. *International Journal of Surgery Case Reports*.

[17] Hafidh M. A., Sheahan P., Khan N. A., Colreavy M., Timon C. (2004). Role of CO_2_ laser in the management of obstructive ectopic lingual thyroids. *The Journal of Laryngology & Otology*.

[18] Puxeddu R., Pelagatti C. L., Nicolai P. (1998). Lingual thyroid: endoscopic management with CO_2_ laser. *American Journal of Otolaryngology—Head and Neck Medicine and Surgery*.

[19] Dasari S. D., Bashetty N. K., Prayaga N. S. M. (2007). Radiofrequency ablation of lingual thyroid. *Otolaryngology—Head and Neck Surgery*.

[20] Wahab S., Khan R. A., Goyal R. (2010). Persistent cough in a lethargic child: watch out for lingual thyroid!. *International Journal of Pediatric Otorhinolaryngology Extra*.

